# Supplementary material for: Roles of WNT6 in Sheep Endometrial Epithelial Cell Cycle Progression and Uterine Glands Organogenesis
Source: Vet Sci. 2021 Dec 9;8(12):316. doi: 10.3390/vetsci8120316 (PMC8708052; doi:10.3390/vetsci8120316)
Supplement: Supplementary file 1 [file Additional file S3.pdf › vetsci-1438271-supplementary/Additional files/Additional file S3.pdf]

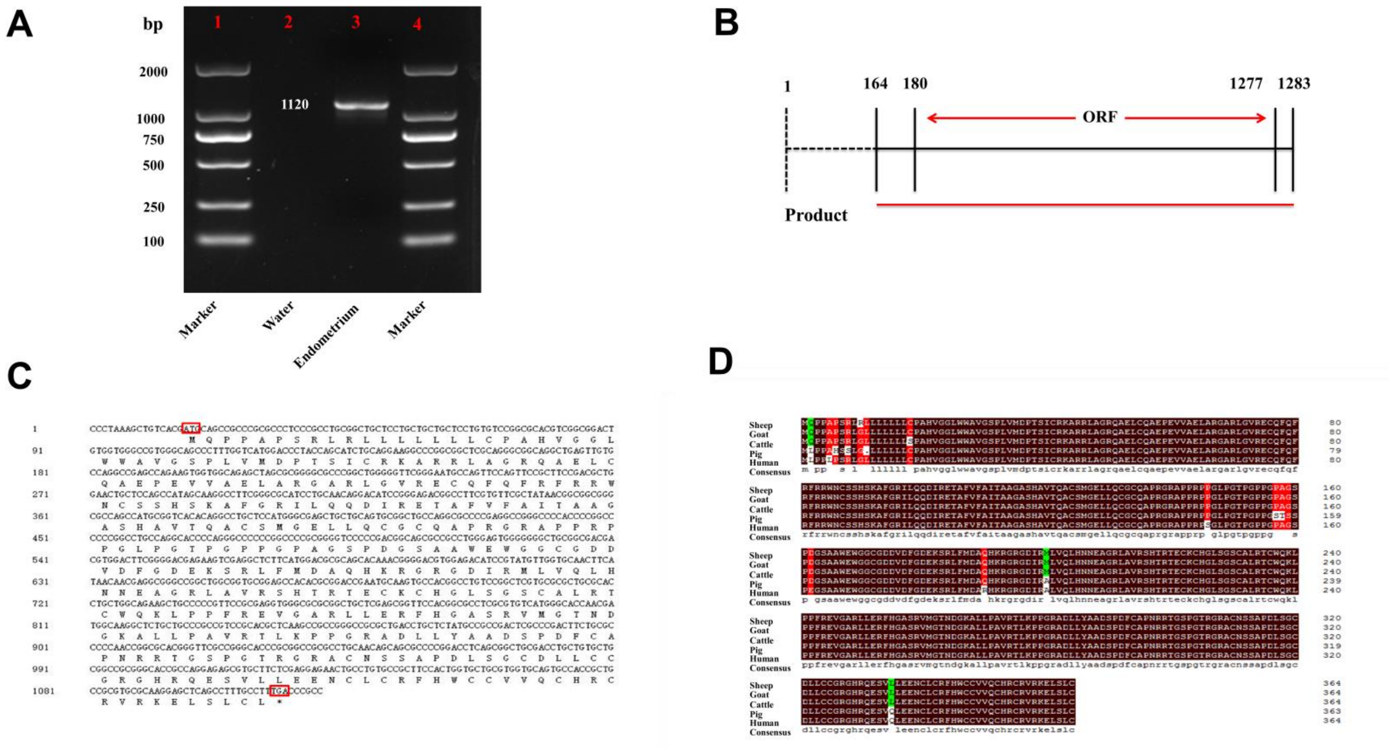

Additional file 3. The CDs cloning and sequence analysis of WNT6. (A) Gel electrophoresis for amplification products of WNT6. (B) Diagram of WNT6 amplification products. (C) Nucleotide sequence and predicted amino acid sequence of sheep WNT6. (D) Alignment of sheep WNT6 with its homologous amino acid from other species. Amino acids sequence homology was showed by brown-shade (100%), red-shade (≥ 75%), and green-shade (≥ 50%) respectively.
