# Supplementary material for: Roles of WNT6 in Sheep Endometrial Epithelial Cell Cycle Progression and Uterine Glands Organogenesis
Source: Vet Sci. 2021 Dec 9;8(12):316. doi: 10.3390/vetsci8120316 (PMC8708052; doi:10.3390/vetsci8120316)
Supplement: Supplementary file 1 [file vetsci-08-00316-s001.zip › vetsci-1438271-supplementary/Additional files/Additional file S4.pdf]

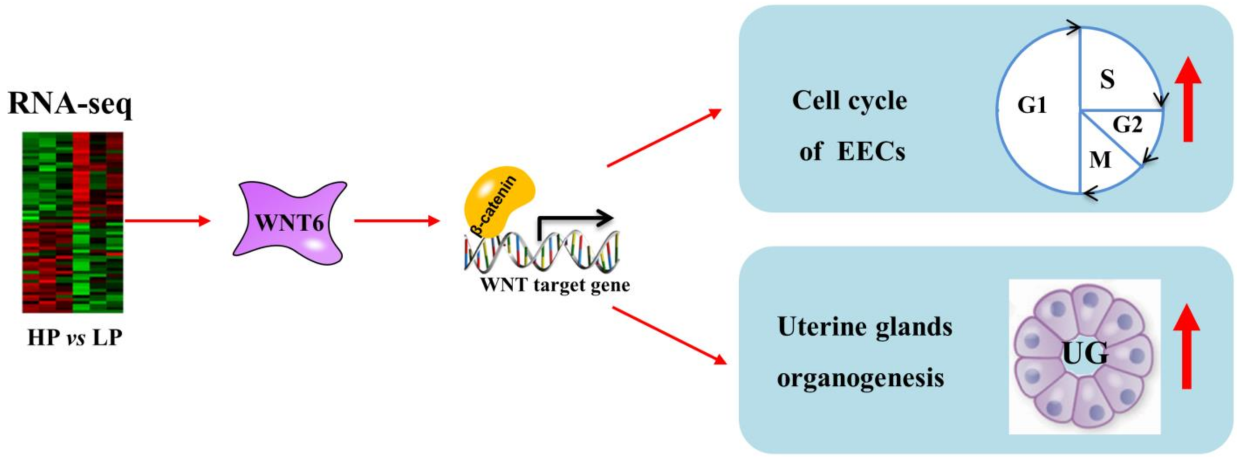

Additional file 4. Roles of WNT6 in EECs cycle progression and UGs organogenesis. EECs, endometrial epithelial cells; UGs, uterine glands; HP, high prolificacy rate Hu sheep; LP, low prolificacy rate Hu sheep.
